# Oropharyngeal cancer mortality in the United States, 1999–2023: a surveillance analysis using CDC WONDER

**DOI:** 10.3389/fonc.2026.1733499

**Published:** 2026-04-01

**Authors:** Weichen Deng, Liren Cao, Zhihai Li

**Affiliations:** 1 School of Medicine, Xiamen University, Xiamen, China; 2 Department of Stomatology, Zunyi Medical University, Zunyi, China; 3 Department of Orthodontics, Guiyang Stomatological Hospital, Guiyang, China; 4 Department of Otorhinolaryngology, Taizhou Municipal Hospital (Taizhou University Affiliated Municipal Hospital), School of Medicine, Taizhou, China

**Keywords:** CDC WONDER database, demographic disparities, epidemiology, Joinpoint regression, mortality trends, oropharyngeal cancer, sociodemographic disparities

## Abstract

**Background:**

Oropharyngeal cancer (OPC) is a major head and neck cancer subtype with shifting U.S. epidemiology. We performed a surveillance-style analysis of OPC mortality trends and demographic/geographic disparities, 1999–2023.

**Methods:**

OPC mortality (1999–2023) was obtained from CDC WONDER. Age-adjusted mortality rates (AAMRs) were computed by category. Joinpoint regression estimated annual percent change (APC) and average annual percent change (AAPC). Pearson correlation assessed associations between AAMR and structural indicators.

**Results:**

From 1999 to 2023, 51,719 OPC-related deaths were recorded. AAMR increased from 0.75 (1999) to 1.17 (2023), with a relatively stable pattern during 1999–2009 (APC = 0.42; 95% CI: −0.47 to 1.32) followed by an increase during 2009–2023 (APC = 3.02; 95% CI: 2.59 to 3.46); the AAPC for 1999–2023 was 1.93 (95% CI: 1.51 to 2.36; *p <* 0.001). Mortality was higher in males (AAMR = 1.44; 95% CI: 1.37 to 1.52) than in females (0.37; 95% CI: 0.34 to 0.41), with increases in both groups (AAPC: 2.16; *p <* 0.001 vs 1.13; *p = 0.001*). By race/ethnicity, overall AAMR was higher among non-Hispanic Black individuals (1.18; 95% CI: 1.03 to 1.32) but declined over time (AAPC = −1.12; *p <* 0.001; 95% CI: −1.76 to −0.47), whereas non-Hispanic White individuals increased (AAPC = 2.92; *p <* 0.001; 95% CI: 2.46 to 3.39) and exceeded non-Hispanic Black individuals in later years. AAMR increased across all U.S. census regions, with higher levels and/or faster increases in the Midwest and South. Nonmetropolitan areas increased faster than metropolitan areas (AAPC: 2.98; *p <* 0.001 vs 1.26; *p <* 0.001). State-level variation was observed; AAMR was inversely correlated with dentist density (r = −0.48, *p = 0.010*) and HPV vaccination coverage (r = −0.44, *p = 0.018*), while the correlation with poverty rate was positive but not statistically significant (r = 0.28, *p = 0.152*).

**Conclusion:**

OPC mortality increased in the United States from 1999 to 2023, with demographic and geographic disparities. These surveillance findings may support public health monitoring and provide a hypothesis-generating basis for future analytic studies.

## 1 Introduction

Oropharyngeal cancer (OPC) is a major subtype of head and neck cancer, and more than 90% of cases are squamous cell carcinomas (1). Common presenting symptoms include sore throat, dysphagia, odynophagia, voice or speech changes, otalgia, and cervical masses. While tobacco, heavy alcohol intake, and human papillomavirus (HPV) infection are well-documented triggers (1), oropharyngeal cancer (OPC) remains a highly dynamic public health challenge. Over recent decades, its incidence has shown a steady upward trajectory (2, 3), climbing roughly 1% annually since the mid-2000s (4). Globally, about 106,400 new cases emerged in 2022 (age-standardized incidence rate (ASIR), 1.1 per 100,000). The disease also claimed 52,305 lives that year, revealing a gender gap in the age-standardized mortality rate (ASMR)—0.9 per 100,000 for men versus just 0.2 for women (5). For 2024, American Cancer Society projections anticipate roughly 58,400 new oral cavity and oropharyngeal diagnoses in the United States, alongside 12,230 fatalities (4, 6). Notably, the epidemiology of OPC is characterized by two concurrent shifts. A clear etiological transition is evident, with the incidence of HPV-associated OPC continuing its sharp increase against a backdrop of declining rates for cancers linked to tobacco and alcohol (7). Concurrently, the demographic profile of patients is evolving. Despite its historical prevalence among older adults, emerging evidence points to a rising incidence in individuals younger than 45 years (8, 9).

Given these evolving epidemiological patterns, ongoing population-level surveillance is undeniably crucial. However, current research leans heavily toward HPV-associated subtypes (10). Recognizing this gap, updated and comprehensive evaluations mapping mortality trends across broad demographic and geographic dimensions remain remarkably scarce. We did not identify prior studies using the CDC WONDER database with mortality data updated through 2023 to describe national trends in OPC-related mortality in the United States. Therefore, we conducted a population-based, surveillance-style analysis of OPC mortality from 1999 to 2023, examining patterns by sex, race/ethnicity, age group, urban–rural status, and census region. In addition, we explored state-level correlations between age-adjusted mortality rates (AAMRs) and selected structural indicators to provide contextual information on geographic variation; these ecological analyses are intended to be hypothesis-generating and do not support causal inference. Overall, this study aims to (1) summarize updated temporal trends in OPC mortality; (2) describe demographic and geographic disparities; and (3) provide an updated epidemiologic reference to support public health monitoring and future analytic research.

## 2 Materials and methods

### 2.1 Study setting and population

This study examined U.S. national mortality trends for oropharyngeal cancer (OPC) from 1999 to 2023. Mortality data were retrieved from the Centers for Disease Control and Prevention (CDC) Wide-ranging Online Data for Epidemiologic Research (WONDER) database, which aggregates death certificate information from all 50 U.S. states and the District of Columbia. We utilized the Multiple Cause of Death (MCOD) public-use files, which contain both the underlying cause of death and contributing causes (11). OPC deaths were identified using International Statistical Classification of Diseases and Related Health Problems, 10th Revision (ICD-10) codes: C01 (base of tongue), C02.4 (lingual tonsil), C05.1 (soft palate, excluding the nasopharyngeal surface), C05.2 (uvula), C09 (tonsil), C10 (oropharynx, excluding tonsil), and C14.2 (Waldeyer ring). ICD-10 codes C14.0 and C14.8 were deemed anatomically ambiguous and were therefore excluded. These case definitions are consistent with those applied in prior population-based investigations of oropharyngeal malignancies (12–14).

Adults were defined as individuals aged ≥ 25 years at death. Records with missing demographic information or CDC-suppressed counts (< 10 deaths) were excluded. Because CDC WONDER provides de-identified, publicly available data, institutional review board approval and informed consent were not required. Data were downloaded in October 2025. This study was reported in accordance with the STROBE guidelines (15).

### 2.2 Data abstraction

Study variables encompassed demographic characteristics, geographic factors, and outcome measures. Demographic variables included sex (male/female), age group (35–44, 45–54, 55–64, 65–74, 75–84, and ≥ 85 years), and race/ethnicity categorized as non-Hispanic White (NH White), non-Hispanic Black (NH Black), Hispanic, and non-Hispanic other (NH Other). Geographic classification followed two established frameworks: the U.S. Census Bureau four-region scheme (Northeast, Midwest, South, and West) and the 2013 National Center for Health Statistics (NCHS) Urban–Rural Classification. Under the NCHS scheme, counties were grouped by metropolitan status as metropolitan (population ≥ 50,000) or nonmetropolitan (population < 50,000) areas (16). Urbanization-stratified mortality rates and trend estimates were limited to 1999–2020 because, within CDC WONDER, the MCOD file that provides county-level NCHS Urban–Rural Classification with compatible population denominators for rate calculation is available through 2020; therefore, AAMR and Joinpoint-derived AAPC by urbanization were computed for 1999–2020, whereas other stratified analyses used the full study period (1999–2023). All these categorizations have been employed in previous CDC WONDER analysis as well (17, 18).

To evaluate the structural drivers of OPC mortality, ecological variables were integrated from multiple federal databases. Dentist density (number of providers per 100,000 population) was sourced from the Area Health Resources Files (AHRF), an integrated database maintained by the Health Resources and Services Administration (HRSA). The average poverty rate was obtained from the U.S. Census Bureau’s Small Area Income and Poverty Estimates (SAIPE) project, utilizing the “All Ages in Poverty” rate based on the official poverty thresholds defined by the Office of Management and Budget (OMB). Additionally, the 2008 female HPV vaccination coverage (adolescents aged 13–17, ≥ 1 dose) was retrieved from the CDC’s National Immunization Survey-Teen (NIS-Teen). Notably, while the 2008 survey relied primarily on landline sampling—which may slightly underrepresent low-income or highly mobile populations prior to the full inclusion of cellular samples in 2011—data were weighted to represent the non-institutionalized adolescent population of the U.S., adjusting for household non-response and the exclusion of households without telephones.

### 2.3 Statistical analysis

The primary outcomes of this study were the crude mortality rate (CMR) and the age-adjusted mortality rate (AAMR) for OPC. Rates were expressed as deaths per 100,000 population. The AAMR was age-standardized to the 2000 U.S. standard population to facilitate comparisons across populations and time periods (19). Age-specific trends were assessed by calculating crude mortality rates (CMR) within defined age strata, as age standardization is not required when analyzing mortality within narrow age groups. All estimates are presented with their corresponding 95% confidence intervals (95% CI). In compliance with CDC confidentiality policies, records with suppressed death counts (< 10) or missing demographic information were excluded from the analysis.

All statistical analyses were performed using RStudio (version 4.4.1). Temporal trends were evaluated through segmented regression analysis using the Joinpoint Regression Program (version 5.4.0; National Cancer Institute, USA). This method identifies significant change points (joinpoints) in a trend by fitting multiple models and determining the optimal number of segments through permutation testing. For each resulting time period, the annual percent change (APC) was calculated to quantify the rate of increase or decrease. The average annual percent change (AAPC) was then derived to summarize the overall trend across the entire study period. The statistical significance of the APC and AAPC estimates was assessed using a two-tailed t-test, with a *p*-value < 0.05 considered significant.

To map the geographic spread and timeline of OPC mortality, we built state-level choropleth maps. These panels track four distinct metrics: total deaths and the age-adjusted mortality rate (AAMR) for 2023, alongside the percent change in raw fatalities between 1999 and 2023 and the overarching AAPC. Percent change was calculated as.


$$\frac{\text{Death}{\text{s}}_{2023}-\text{Death}{\text{s}}_{1999}}{\text{Death}{\text{s}}_{1999}}\times 100\%$$

**Figure 1:**
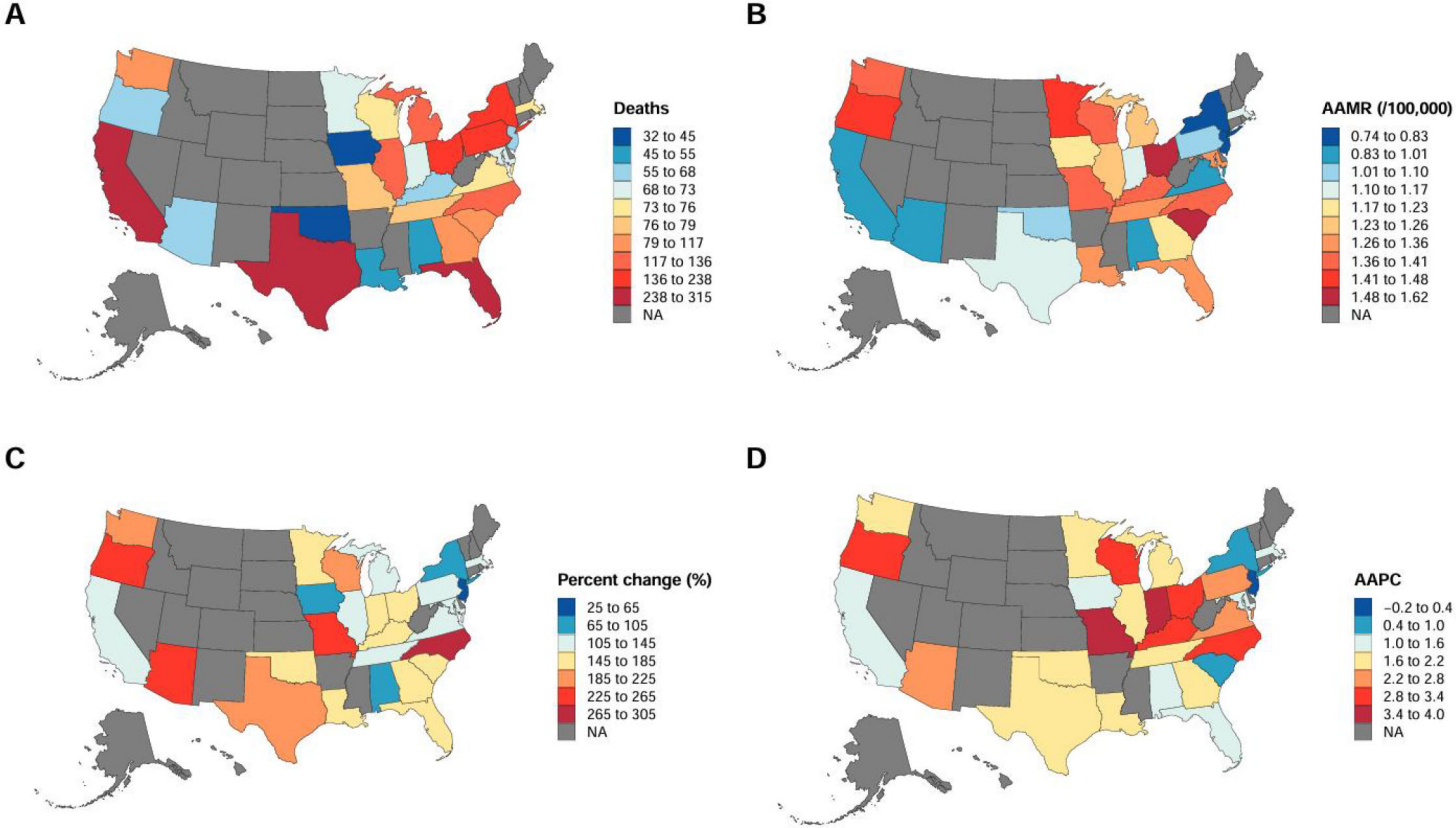
State-level variations in OPC mortality, 1999–2023. **(A)** Total deaths in 2023. **(B)** Age-adjusted mortality rate (AAMR) per 100,000 population in 2023. **(C)** Percent change in deaths from 1999 to 2023. **(D)** Average annual percent change (AAPC) over the study period.

AAPC estimates were obtained from Joinpoint regression outputs. When formatting the map legends, we grouped the 2023 deaths and AAMR data into decile-based quantiles. Conversely, the percent change and AAPC relied on predefined interval breaks to better clarify regional contrasts. We handled the actual spatial rendering in Rstudio using the usmap package, applying ggplot2 for styling, and arranged the outputs into a 2 × 2 panel. Notably, the analysis in Section 3.6 utilizes the overall AAMR across the entire study period, while **Figure 1B** illustrates the AAMR for the year 2023.

To explore state-level structural factors associated with OPC mortality, Pearson correlation analyses were conducted in Rstudio. The dependent variable was the five-year average AAMR (2019–2023), which helped smooth out routine annual fluctuations in mortality estimates. Independent variables included the mean poverty rate and dentist density (per 100,000 population) from 2010 to 2015. The poverty rate was incorporated with an approximate 10-year lag to account for the latency of carcinogenesis and the cumulative impact of socioeconomic disadvantage on health outcomes. Dentist density served as a surrogate for healthcare accessibility and screening capacity. Additionally, state-level female HPV vaccination rates from 2008 were included as a proxy for early public health awareness and policy performance. The normality of all variables was confirmed using Q-Q plots. Associations were visualized using scatter plots with fitted linear regression lines, generated with the ggplot2 package.

## 3 Results

### 3.1 Temporal trends for OPC-related AAMR

**Figure 2:**
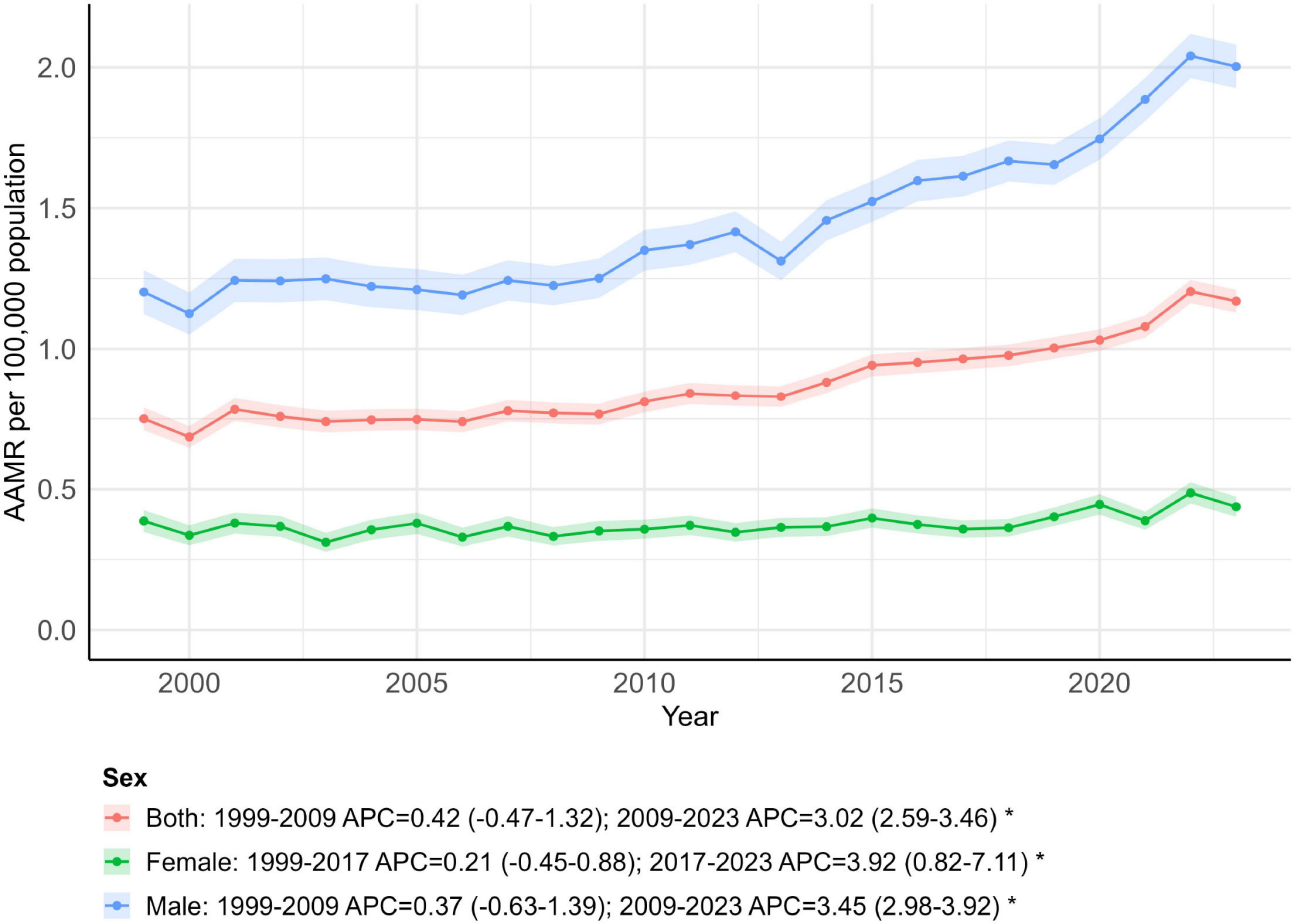
Trends in age-adjusted mortality rates by sex, 1999–2023. * indicates that the annual percent change (APC) is significantly different from zero (*p* < 0.05).

A total of 51,719 OPC-related deaths were documented in the United States from 1999 to 2023 (**Supplementary Table 1**). AAMR increased slightly from 0.75 in 1999 to 0.77 in 2009 (APC = 0.42; 95% CI: −0.47 to 1.32). From 2009 to 2023, AAMR increased from 0.77 to 1.17 (APC = 3.02; 95% CI: 2.59 to 3.46). Overall, the trend shifted from a relatively stable pattern in the earlier period to a sustained increase after 2009, with an AAPC of 1.93 (95% CI: 1.51 to 2.36; *p <* 0.001) over the full study period (**Figure 2**; **Supplementary Tables 2**, **3**).

### 3.2 OPC-related AAMR stratified by sex

From 1999 to 2023, there were 39,504 deaths related to OPC among males and 12,079 among females in the U.S. (**Supplementary Table 1**). The overall AAMR was higher in males (1.44; 95% CI: 1.37 to 1.52) than in females (0.37; 95% CI: 0.34 to 0.41). Among males, the AAMR remained relatively stable between 1999 and 2009 (APC = 0.37; 95% CI: –0.63 to 1.39), followed by an increase from 2009 to 2023 (APC = 3.45; 95% CI: 2.98 to 3.92). In females, mortality rates were relatively stable between 1999 and 2017 (APC = 0.21; 95% CI: −0.45 to 0.88) and then increased from 2017 to 2023 (APC = 3.92; 95% CI: 0.82 to 7.11) (**Figure 2**; **Supplementary Tables 2**, **3**). Overall, increasing trends in OPC mortality were observed for both sexes, with the joinpoint indicating a steeper rise occurring earlier among males than among females. Over the whole 1999–2023 study period, the AAPC was 2.16 (95% CI: 1.68 to 2.63; *p <* 0.001) for males and 1.13 (95% CI: 0.27 to 1.99; *p = 0.001*) for females, consistent with an overall increase in OPC mortality in both groups (**Supplementary Table 4**).

### 3.3 OPC-related AAMR stratified by race

**Figure 3:**
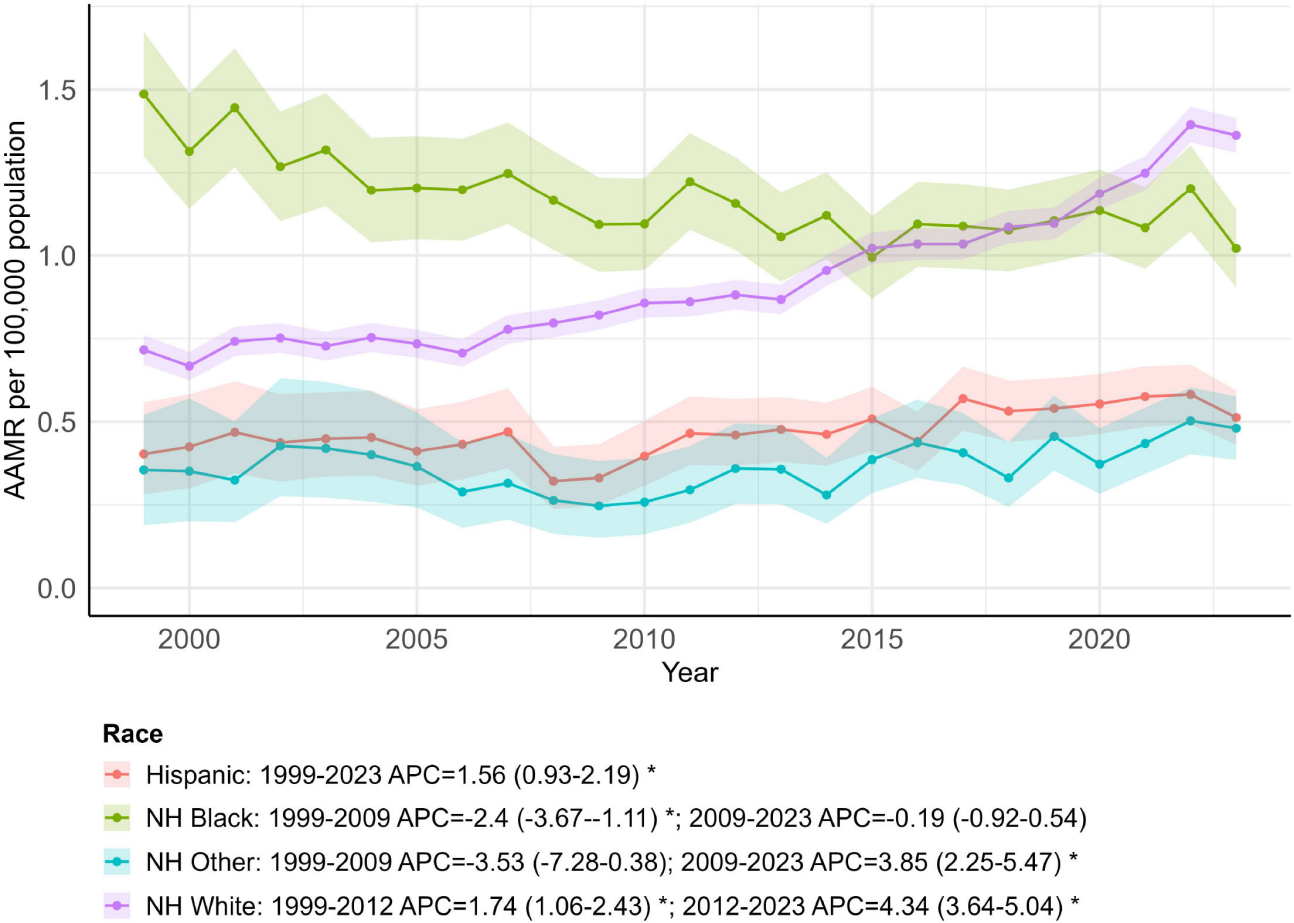
Trends in age-adjusted mortality rates by race, 1999–2023. * indicates that the annual percent change (APC) is significantly different from zero (*p* < 0.05).

From 1999 to 2023, the largest number of OPC-related deaths occurred among non-Hispanic (NH) White individuals (41,196 deaths), whereas the fewest occurred among NH Other groups (1,088 deaths) in the United States (**Supplementary Table 5**). The highest overall AAMR occurred in the non-Hispanic Black population (1.18; 95% CI: 1.03 to 1.32). Mortality in this group declined overall, with a decrease from 1999 to 2009 (APC = −2.40; 95% CI: −3.67 to −1.11) followed by a relatively stable pattern thereafter (APC = −0.19; 95% CI: −0.92 to 0.54). In contrast, NH White individuals had a lower overall AAMR (0.92; 95% CI: 0.88 to 0.97) and showed increasing mortality over time, with an increase during 1999–2012 (APC = 1.71; 95% CI: 1.06 to 2.43) and a steeper increase during 2012–2023 (APC = 4.34; 95% CI: 3.64 to 5.04). Consistent with these divergent trends, AAMR among NH White individuals became higher than that of the NH Black population in the later years of the study period. The overall AAMRs were lower among Hispanic individuals (0.47; 95% CI: 0.36 to 0.57) and NH Other (0.36; 95% CI: 0.24 to 0.49), with relatively modest long-term changes over the study period (**Figure 3**; **Supplementary Tables 6**, **7**). AAMR increased more rapidly among non-Hispanic White individuals (AAPC = 2.92; 95% CI: 2.46 to 3.39; *p <* 0.001), showed a more modest increase among Hispanics (AAPC = 1.56; 95% CI: 0.93 to 2.19; *p <* 0.001), and declined among non-Hispanic Black individuals (AAPC = –1.12; 95% CI: –1.76 to –0.47; *p <* 0.001) over the study period (**Supplementary Table 8**). Overall, racial and ethnic disparities in OPC mortality were observed across the study period.

### 3.4 OPC-related AAMR stratified by census region

**Figure 4:**
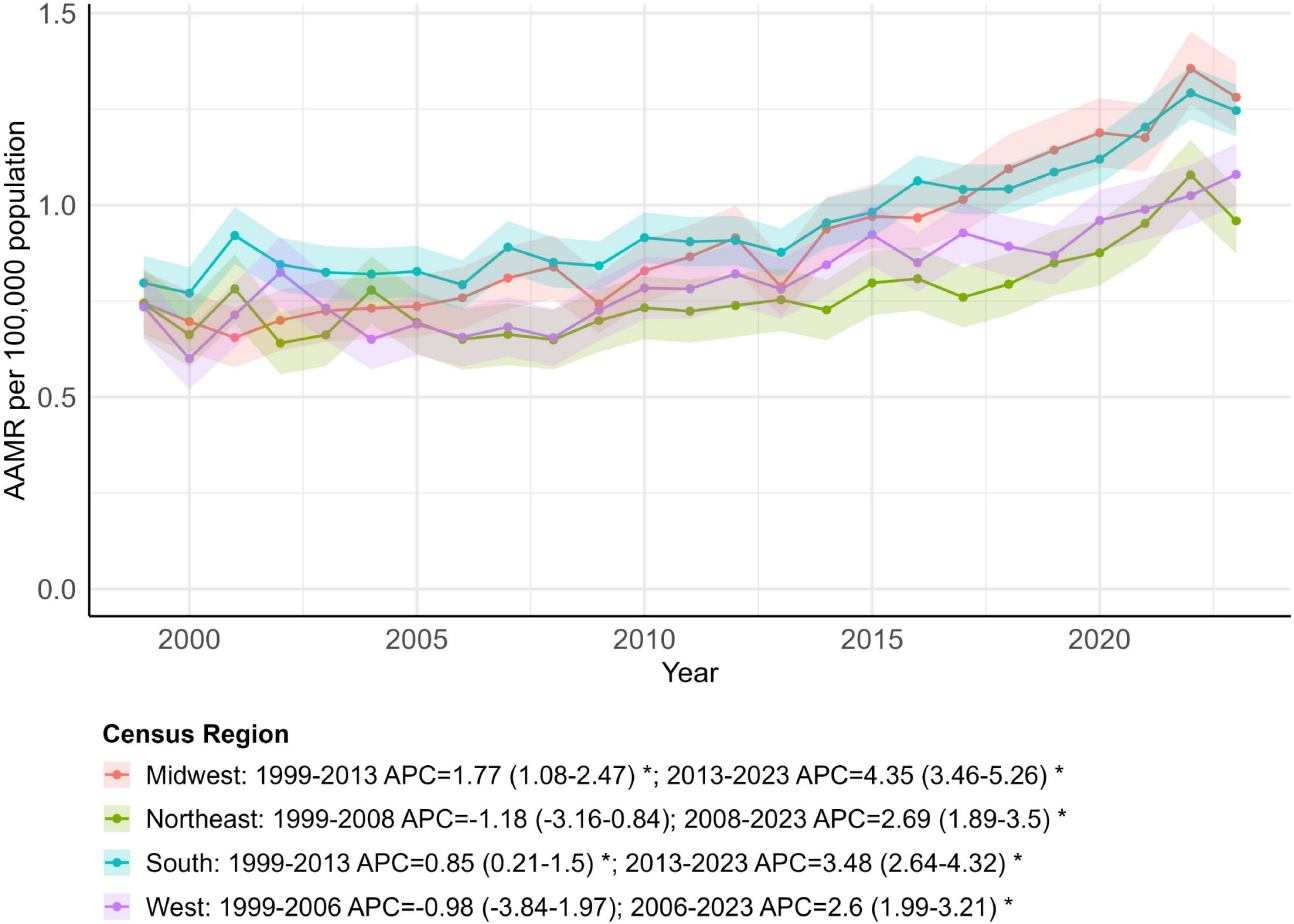
Trends in age-adjusted mortality rates by census region, 1999–2023. * indicates that the annual percent change (APC) is significantly different from zero (*p* < 0.05).

The South had the largest number of OPC-related deaths (20,883), followed by the Midwest (11,874), West (10,426), and Northeast (8,536) (**Supplementary Table 9**). This distribution was consistent with regional AAMRs, which were higher in the South (0.95; 95% CI: 0.89 to 1.02) and Midwest (0.91; 95% CI: 0.82 to 0.99) and lower in the West (0.81; 95% CI: 0.73 to 0.89) and Northeast (0.77; 95% CI: 0.68 to 0.85). Joinpoint analyses indicated increasing AAMR trends across all four U.S. regions over time, with region-specific joinpoints suggesting a shift from relatively modest changes in earlier years to faster increases in more recent periods. The Midwest showed an increase between 1999 and 2013 (APC = 1.77; 95% CI: 1.08 to 2.47), followed by a steeper increase from 2013 to 2023 (APC = 4.35; 95% CI: 3.46 to 5.26). The South also increased between 1999 and 2013 (APC = 0.85; 95% CI: 0.21 to 1.50) and increased more rapidly from 2013 to 2023 (APC = 3.48; 95% CI: 2.64 to 4.32) (**Figure 4**; **Supplementary Tables 10**, **11**). From an AAPC perspective, the Midwest (AAPC = 2.84; 95% CI: 2.32 to 3.36; *p <* 0.001) and the South (AAPC = 1.94; 95% CI: 1.46 to 2.42; *p<* 0.001) had the largest AAPCs in OPC mortality among the four regions (**Supplementary Table 12**).

### 3.5 OPC-related AAMR stratified by urban–rural status

**Figure 5:**
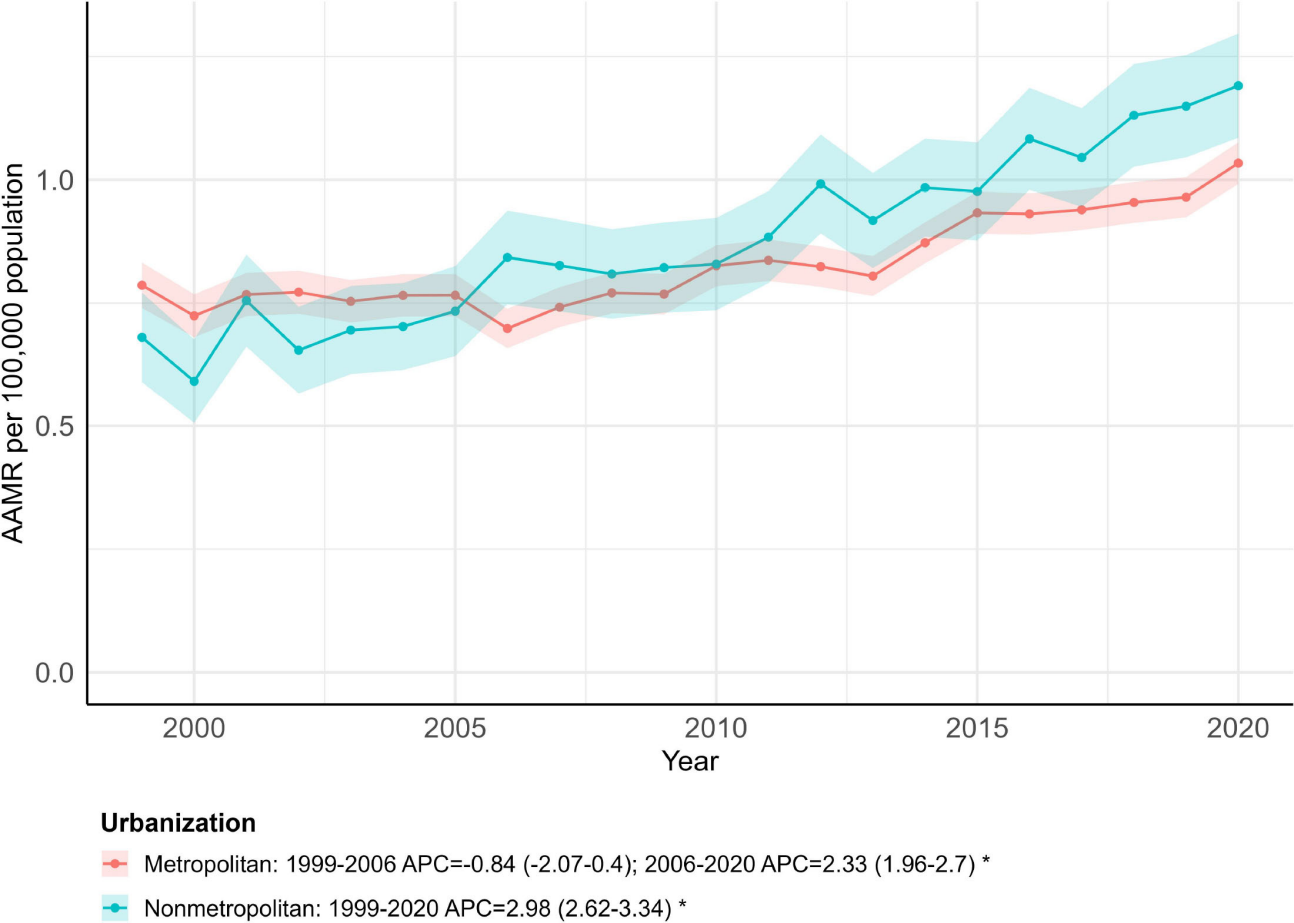
Trends in age-adjusted mortality rates by urbanization, 1999–2020. * indicates that the annual percent change (APC) is significantly different from zero (*p* < 0.05).

Between 1999 and 2020, a total of 34,379 OPC-related deaths were recorded in metropolitan areas, while 7,479 deaths occurred in nonmetropolitan regions of the United States (**Supplementary Table 13**). The overall AAMR was slightly higher in nonmetropolitan counties (0.88; 95% CI: 0.78 to 0.97) than in metropolitan areas (0.83; 95% CI: 0.79 to 0.87). In metropolitan areas, AAMR decreased from 1999 to 2006 (APC = −0.84; 95% CI: −2.07 to 0.40) and then increased from 2006 to 2020 (APC = 2.33; 95% CI: 1.96 to 2.70). The AAMR in nonmetropolitan regions steadily increased between 1999 and 2020 (APC = 2.98; 95% CI: 2.62 to 3.34) (**Figure 5**; **Supplementary Tables 14**, **15**). Over the full period, AAPCs were 1.26 (95% CI: 0.81 to 1.71; *p <* 0.001) in metropolitan areas and 2.98 (95% CI: 2.62 to 3.34; *p <* 0.001) in nonmetropolitan areas (**Supplementary Table 16**). Overall, AAMR increased in both settings, with faster increases observed in nonmetropolitan areas.

### 3.6 OPC-related AAMR stratified by state

From 1999 to 2023, the largest numbers of oropharyngeal cancer–related deaths were reported in California (5,134), Florida (4,366), and Texas (3,741), whereas the smallest numbers occurred in Iowa (541), Oklahoma (591), and Alabama (827) (**Supplementary Table 17**). State-level variation in OPC mortality was observed. Several states exhibited higher overall AAMRs (AAMR > 1.0), including Washington, Oregon, Ohio, Kentucky, Florida, and North Carolina. States with higher increases over time (AAPC > 2.8) included Indiana, Missouri, Kentucky, Ohio, Oregon, North Carolina, and Wisconsin. Ohio and Oregon had both high AAMRs and high AAPCs, indicating comparatively higher mortality levels and faster increases during the study period. Many of these states were located in the Midwest and the South, consistent with the regional patterns described in Section 3.4. In contrast, California, New York, Arizona, and New Jersey consistently exhibited lower AAMRs, while California, South Carolina, New York, and New Jersey showed relatively slower temporal changes. California, New York, and New Jersey had both lower AAMRs and relatively modest increases during the study period (**Figure 1**; **Supplementary Tables 18**, **19**).

**Figure 6:**
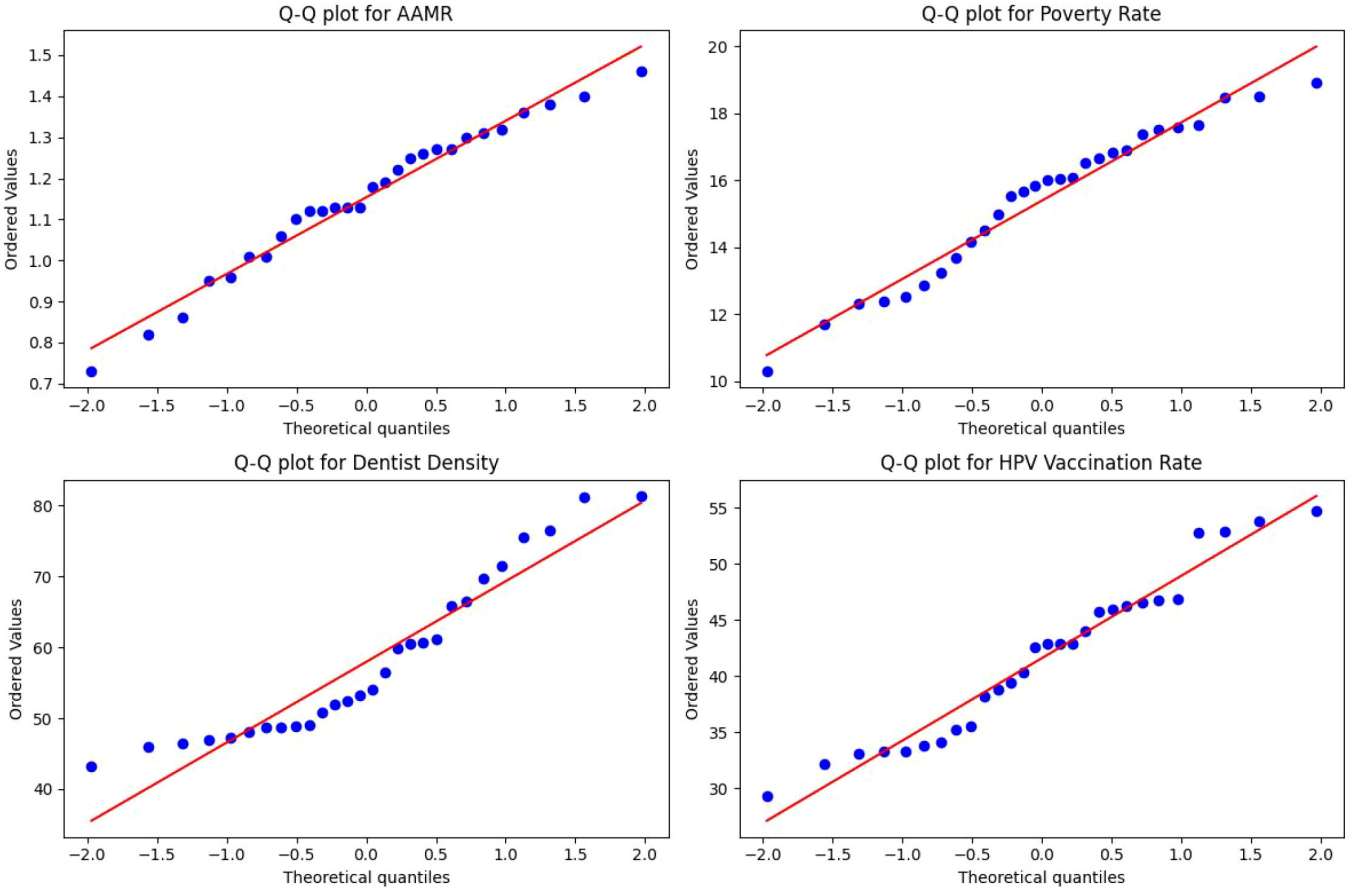
Quantile-Quantile (Q-Q) plots for normality assessment of AAMR and independent variables.

**Figure 7:**
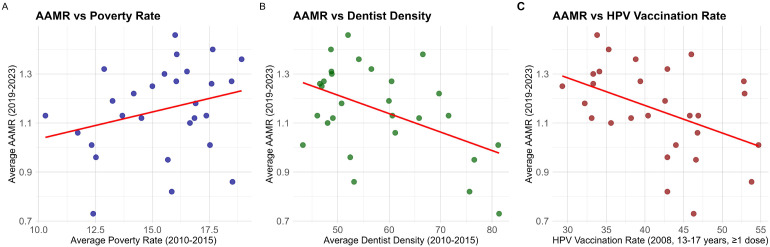
Correlations between state-level OPC AAMR and selected structural factors. **(A)** Correlation between average AAMR (2019–2023) and average poverty rate (2010–2015). **(B)** Correlation between average AAMR (2019–2023) and dentist density (per 100,000 population, 2010–2015). **(C)** Correlation between average AAMR (2019–2023) and HPV vaccination rate (2008, females aged 13–17 years, >1 dose).

Pearson correlation analyses were performed to assess the associations between state-level OPC AAMR and socioeconomic, healthcare, and preventive indicators. The distributions of variables were visually assessed using Q–Q plots (**Figure 6**) to support the use of parametric correlation methods. As illustrated in **Figure 7**, AAMR was inversely correlated with dentist density (r = −0.48, *p = 0.010*) and with HPV vaccination coverage (r = −0.44, *p = 0.018*). In contrast, AAMR showed a positive but non-significant correlation with the average poverty rate (r = 0.28, *p = 0.152*). These ecological associations do not imply causality and should be interpreted as contextual, hypothesis-generating findings. The underlying data for **Figure 7** are provided in **Supplementary Table 20**.

### 3.7 OPC-related mortality stratified by age group (CMR)

OPC-related mortality varied across age groups during 1999–2023, with 1,048 deaths recorded among individuals aged 35–44 years, 7,071 among those aged 45–54 years, 15,599 among those aged 55–64 years, 14,940 among those aged 65–74 years, 9,364 among those aged 75–84 years, and 3,644 among those aged ≥85 years (**Supplementary Table 21**).

**Figure 8:**
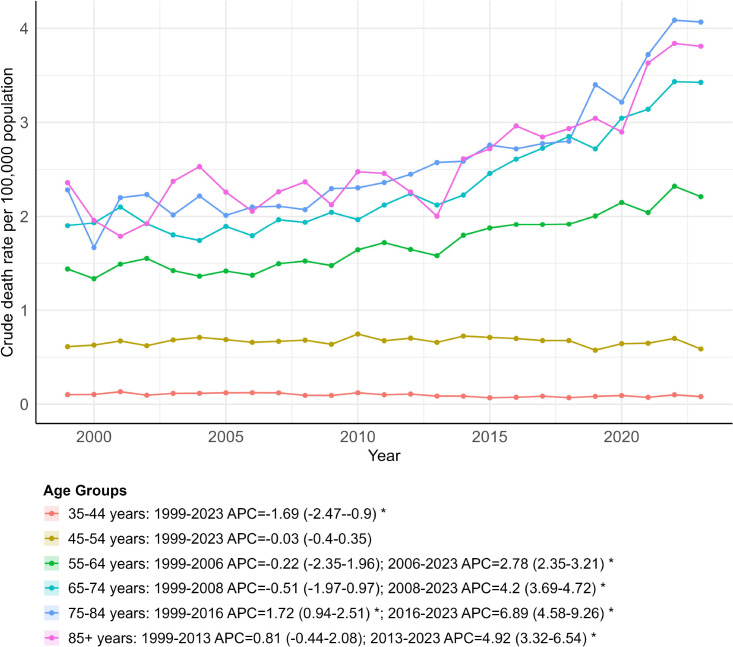
Trends in crude mortality rates by age group, 1999–2023. * indicates that the annual percent change (APC) is significantly different from zero (*p* < 0.05).

The crude mortality rate (CMR) declined in the 35–44 age group from 1999 to 2023 (APC = –1.69; 95% CI: –2.47 to –0.90), whereas that in the 45–54 age group remained stable (APC = –0.03; 95% CI: –0.40 to 0.35). In contrast, individuals aged 55 years and older exhibited an upward tendency in CMR over the study period. For example, among those aged 75–84, CMR rose between 1999 and 2016 (APC = 1.72; 95% CI: 0.94 to 2.51) and then increased more rapidly during 2016–2023 (APC = 6.89; 95% CI: 4.58 to 9.26) (**Figure 8**; **Supplemental Table 22**, **Supplemental Table 23**). Over the full study period, AAPCs were −1.69 (95% CI: −2.47 to −0.90; *p* < 0.001) for ages 35–44 years, −0.03 (95% CI: −0.40 to 0.35; *p = 0.875*) for ages 45–54 years, 1.90 (95% CI: 1.23–2.56; *p* < *0.001*) for ages 55–64 years, 2.41 (95% CI: 1.80 to 3.02; *p* < *0.001*) for ages 65–74 years, 3.20 (95% CI: 2.39 to 4.02; *p* < *0.001*) for ages 75–84 years, and 2.50 (95% CI: 1.57 to 3.44; *p* < *0.001*) for ages ≥ 85 years (Supplemental Table 24), reflecting larger increases in mortality in older age groups.

## 4 Discussion

### 4.1 Overall temporal trend and potential etiologic context

We observed a continued increase in the overall AAMR of oropharyngeal cancer, with a more pronounced upward trend after 2009. As a descriptive, population-based analysis, this study documents these trends and disparities but cannot establish their etiologic causes. This pattern is consistent with recent reports (20), and similar increases in OPC mortality have also been described in other countries (21). Historically, tobacco and alcohol use have been major risk factors for OPC; however, adult smoking prevalence in the United States has declined steadily since the 1980s (22, 23), and population-level alcohol consumption has remained stable or declined slightly in recent years (24). Against this background, the observed mortality trends may be compatible with the evolving epidemiology of HPV-associated OPC reported in prior studies. Since the 1980s, the incidence of HPV-positive OPC has increased, whereas HPV-negative OPC has declined; for example, between 1988 and 2004, HPV-positive OPC incidence increased substantially while HPV-negative incidence decreased (25), a pattern that parallels long-term reductions in smoking prevalence. It is reasonable to assume that differences in prognosis and treatment alter how incidence trends translate into mortality over time. Clinically, HPV-positive OPC often yields better survival than HPV-negative disease (26). Meanwhile, progress in radiotherapy and supportive care has improved outcomes in specific settings (27). Together with the long latency of HPV-related carcinogenesis (28), these factors could contribute to a temporal disconnect in which shifts in incidence are observed earlier, while changes in mortality become more apparent later; however, such mechanisms cannot be directly evaluated in the present descriptive mortality analysis. Consistent with this interpretation, multiple studies have documented increasing incidence and disease burden of HPV-related OPC globally over the past two decades (29–33), including a reported tripling of incidence in the United States between 2000 and 2017 (32).

### 4.2 Sex differences

The overall AAMR among males was approximately four times that of females, with an earlier onset of increase. The observed disparity is consistent with reported differences in established risk factors. First, it aligns with the historically higher prevalence of tobacco use and alcohol consumption among men (34). Second, it mirrors epidemiologic evidence of higher oral HPV prevalence and incidence of HPV-positive OPC in men (35–37). The pattern is also compatible with proposed biological differences in HPV infection dynamics or immune response by sex (28, 38). Furthermore, as occupational exposures linked to OPC risk (39, 40) are more common in male-dominated industries, differential exposure may also contribute. Although routine HPV vaccination was recommended earlier for females (2006) than for males (2011) (41, 42), the long latency between HPV infection, cancer development, and mortality suggests that any vaccination-related effects on mortality may not yet be fully reflected within the current study period. Overall, these observations should be interpreted cautiously and viewed as hypothesis-generating rather than mechanistic explanations. From a clinical and public health perspective, the observed sex-specific patterns may warrant continued attention to symptom awareness, timely evaluation, and prevention efforts, including ongoing gender-neutral HPV vaccination strategies (43) and education to improve awareness of HPV-related OPC, particularly among men (44).

### 4.3 Racial/ethnic differences

Although the overall AAMR was higher among non-Hispanic (NH) Black individuals during much of the study period, mortality in this group declined over time. This decline is consistent with documented reductions in smoking prevalence among Black populations since the late 20th century (45, 46), although causal attribution cannot be made from the present descriptive analysis. In contrast, AAMR increased among NH White individuals, contributing to a crossover pattern in which mortality among NH Whites became higher than that among NH Blacks in the later years of the study period. As tobacco-attributable OPC declines, these divergent trajectories may be compatible with an increasing relative contribution of HPV-associated disease, particularly among NH White populations. Prior studies have reported a higher prevalence of HPV-positive OPC among White individuals compared with Black individuals (32, 47). Differences in HPV exposure and infection dynamics—including factors related to sexual behavior and other social determinants—have been proposed as potential contributors (48–52), but these explanations cannot be evaluated directly within the current mortality-based dataset. Overall, the observed crossover pattern highlights evolving population-level risk profiles and suggests that prevention and monitoring efforts may benefit from considering both traditional risk factors (e.g., tobacco and alcohol) and HPV-related disease dynamics in future analytic work.

### 4.4 State-level variation and structural correlates

State-level analyses showed substantial variation in OPC mortality levels and temporal change. Patterns of higher mortality levels and/or faster increases were more commonly observed in parts of the Midwest and South, consistent with the broader regional results and prior population-based evidence on geographic and sociodemographic differences in head and neck cancer outcomes and care access (53–55). To provide contextual insight into geographic variation, we examined ecological correlations between state-level AAMR and selected structural indicators. In these analyses, higher dentist density and higher HPV vaccination coverage were inversely correlated with AAMR, whereas poverty rate showed a positive but non-significant correlation. These associations are ecological and do not imply causality. Dentist density may serve as a proxy for access to preventive oral health services and broader healthcare capacity. Adolescent female HPV vaccination coverage in 2008 may reflect differences in public health program implementation and preventive orientation; however, given the long latency between HPV infection, cancer development, and cancer-related death (28), vaccination-related effects on mortality would not be expected to be fully reflected within the current mortality time horizon. Accordingly, vaccination coverage is best interpreted here as a contextual indicator rather than a direct determinant of contemporary mortality differences. The non-significant association between poverty rate and AAMR should be interpreted cautiously. With a limited sample size at the state level and substantial heterogeneity, statistical power may be insufficient to detect modest associations, and a p-value above 0.05 does not establish the absence of a relationship. Ecological analyses are also susceptible to confounding by unmeasured factors (e.g., smoking and alcohol patterns, healthcare utilization, stage at diagnosis, treatment access, and HPV-attributable fraction) and collinearity among structural indicators (56–60). Therefore, these findings should be viewed as hypothesis-generating and supportive of further individual-level or longitudinal studies. Overall, these state-level patterns and ecological correlations support the value of surveillance-oriented analyses for identifying where mortality is higher or increasing faster, and for highlighting structural domains—such as preventive service access and public health program implementation—that may warrant closer monitoring and future analytic investigation (56–60).

### 4.5 Urban–rural differences

The faster increase in AAMR observed in nonmetropolitan areas in recent years may indicate a widening urban–rural difference in OPC mortality. Although this study is descriptive and cannot determine causal drivers, the observed pattern highlights the importance of continued monitoring of rural populations and of considering structural factors related to access to preventive and diagnostic services. In resource-constrained environments, expanding primary and dental care access provides a practical baseline for public health planning. Sustaining standard tobacco control and delivering preventive measures like HPV vaccination are also logical steps. While one might expect these interventions to help, their actual impact on mortality disparities remains unclear; future analytic studies must evaluate this directly.

### 4.6 Age-group differences

Current data indicate that OPC mortality is increasingly concentrated among patients aged ≥ 55, with the most rapid recent rises observed in the oldest age groups. Several factors may contribute to this pattern. Cohort-related differences in historical exposure to tobacco and alcohol have been proposed as potential contributors, although such explanations cannot be tested directly in the present analysis. Beyond lifetime exposures, differences in treatment patterns by age are also relevant. Prior work suggests that a substantial proportion of older OPC patients may be undertreated. For instance, individuals aged 65 and above forgo initial therapy more often than younger patients (24.5% versus 19.0%) (61). These disparities likely reflect a combination of clinical and socioeconomic considerations, including comorbidity burden, functional status (e.g., Karnofsky Performance Status), and the balance between expected therapeutic benefit and quality of life in older adults (62). Additionally, older patients may face greater financial toxicity during cancer care (63).

From a clinical and public health perspective, these findings highlight the need for continued attention to the timely evaluation of persistent oropharyngeal symptoms in older adults, particularly in primary care and dental settings. Practical approaches, such as enhancing symptom awareness and streamlining pathways to diagnostic assessment, could be considered. However, the feasibility and effectiveness of any formal screening-oriented strategies would require evaluation in future studies. To optimize management for this population, emerging adaptive risk-stratification technologies—such as liquid biopsies and metabolic imaging—offer a promising path toward precision oncology (64).

### 4.7 Strengths and limitations

#### 4.7.1 Strengths

This study includes 51,719 OPC-related deaths recorded between 1999 and 2023, providing a large sample size and long observation period for characterizing temporal and geographic variation in mortality. We used the most recent CDC WONDER mortality release, a nationally representative data source widely used for U.S. surveillance analyses. In addition to descriptive trend analyses, we examined state-level correlations with selected socioeconomic and healthcare indicators to provide contextual, hypothesis-generating insight into structural variation across states. Overall, these results may support ongoing public health monitoring and help prioritize areas for future analytic investigation.

#### 4.7.2 Limitations

Nonetheless, this study has several limitations. First and foremost, its ecological and descriptive design precludes causal inference; population-level associations are subject to ecological fallacy and should be interpreted as hypothesis-generating rather than as evidence of individual-level risk or mechanisms. Second, the mortality data do not distinguish HPV-positive from HPV-negative OPC, precluding subtype-specific trend analyses. Third, CDC WONDER suppresses small state-year cell counts (e.g., fewer than 10 deaths) for privacy protection, leading to incomplete data that may affect geographic and subgroup comparisons; as a result, some racial/ethnic groups with small numbers and the 25–34-year age group could not be reliably analyzed. Fourth, the lack of individual-level information—such as smoking and alcohol history, HPV status, stage at diagnosis, treatment, comorbidity burden, insurance coverage, and socioeconomic indicators—limits adjustment for confounding and constrains interpretation of observed disparities. Fifth, cause-of-death coding based on death certificates may be subject to misclassification and variation in reporting practices. Finally, our state-level correlation analyses were exploratory, based on a limited number of observational units and structural indicators measured within specific time windows; therefore, limited statistical power, potential collinearity, and multiple comparisons without formal adjustment warrant cautious interpretation of these results.

## 5 Conclusion

Based on U.S. national mortality data from 1999 to 2023, this descriptive study characterizes trends and disparities in oropharyngeal cancer (OPC) mortality. Overall age-adjusted mortality increased during the study period, with a more pronounced upward trend after 2009. Mortality patterns varied across demographic and geographic groups, with higher levels and/or faster increases observed among males, non-Hispanic White individuals, residents of the South and Midwest, nonmetropolitan counties, and older adults. This ecological analysis identifies population-level disparities but cannot determine their underlying causes. In conclusion, this work provides an updated surveillance profile of OPC mortality in the United States. These findings may support ongoing public health monitoring and serve as a hypothesis-generating foundation for future analytic studies examining factors that contribute to observed disparities.

## Data Availability

Publicly available datasets were analyzed in this study. This data can be found here: https://wonder.cdc.gov/.
