# Supplementary material for: Oropharyngeal cancer mortality in the United States, 1999–2023: a surveillance analysis using CDC WONDER
Source: Front Oncol. 2026 Apr 1;16:1733499. doi: 10.3389/fonc.2026.1733499 (PMC13079036; doi:10.3389/fonc.2026.1733499)
Supplement: Supplementary file 1 [file Table1.docx]

**Table 1. Number of Oropharyngeal Cancer Deaths by Sex and Year, 1999–2023.**

| **Year** | **Overall** | **Women** | **Men** | **Population** |
| --- | --- | --- | --- | --- |
| 1999 | 1341 | 391 | 950 | 180408769 |
| 2000 | 1254 | 360 | 894 | 181984640 |
| 2001 | 1441 | 408 | 1033 | 184305128 |
| 2002 | 1432 | 393 | 1039 | 186208028 |
| 2003 | 1433 | 349 | 1084 | 188090429 |
| 2004 | 1477 | 397 | 1080 | 190205384 |
| 2005 | 1508 | 417 | 1091 | 192551384 |
| 2006 | 1498 | 391 | 1107 | 195019359 |
| 2007 | 1620 | 420 | 1200 | 197403777 |
| 2008 | 1658 | 417 | 1241 | 199795090 |
| 2009 | 1700 | 420 | 1280 | 202107016 |
| 2010 | 1850 | 445 | 1405 | 203891983 |
| 2011 | 1934 | 475 | 1459 | 206592936 |
| 2012 | 1992 | 450 | 1542 | 208826037 |
| 2013 | 1951 | 469 | 1482 | 211085314 |
| 2014 | 2177 | 499 | 1678 | 213809280 |
| 2015 | 2339 | 549 | 1790 | 216553817 |
| 2016 | 2449 | 545 | 1904 | 218641417 |
| 2017 | 2532 | 532 | 2000 | 221447331 |
| 2018 | 2620 | 542 | 2078 | 223311190 |
| 2019 | 2726 | 607 | 2119 | 224981167 |
| 2020 | 2926 | 649 | 2277 | 226635013 |
| 2021 | 3050 | 596 | 2454 | 228238412 |
| 2022 | 3436 | 728 | 2708 | 229508599 |
| 2023 | 3375 | 660 | 2715 | 231529762 |
| Total | 51719 | 12109 | 39610 | 5163131262 |

**Table 2. Age-Adjusted Mortality Rates by Sex and Year, 1999–2023.**

| **Year** | **Men** | **Women** | **Overall** |
| --- | --- | --- | --- |
| 1999 | 1.20(1.12–1.28) | 0.39(0.35–0.43) | 0.75(0.71–0.79) |
| 2000 | 1.13(1.05–1.20) | 0.34(0.30–0.37) | 0.69(0.65–0.72) |
| 2001 | 1.24(1.17–1.32) | 0.38(0.34–0.42) | 0.78(0.74–0.82) |
| 2002 | 1.24(1.17–1.32) | 0.37(0.33–0.40) | 0.76(0.72–0.80) |
| 2003 | 1.25(1.17–1.32) | 0.31(0.28–0.34) | 0.74(0.70–0.78) |
| 2004 | 1.22(1.15–1.30) | 0.36(0.32–0.39) | 0.75(0.71–0.78) |
| 2005 | 1.21(1.14–1.28) | 0.38(0.34–0.42) | 0.75(0.71–0.79) |
| 2006 | 1.19(1.12–1.26) | 0.33(0.30–0.36) | 0.74(0.70–0.78) |
| 2007 | 1.24(1.17–1.32) | 0.37(0.33–0.40) | 0.78(0.74–0.82) |
| 2008 | 1.23(1.16–1.29) | 0.33(0.30–0.36) | 0.77(0.73–0.81) |
| 2009 | 1.25(1.18–1.32) | 0.35(0.32–0.39) | 0.77(0.73–0.80) |
| 2010 | 1.35(1.28–1.42) | 0.36(0.32–0.39) | 0.81(0.77–0.85) |
| 2011 | 1.37(1.30–1.44) | 0.37(0.34–0.41) | 0.84(0.80–0.88) |
| 2012 | 1.42(1.34–1.49) | 0.35(0.31–0.38) | 0.83(0.80–0.87) |
| 2013 | 1.31(1.24–1.38) | 0.36(0.33–0.40) | 0.83(0.79–0.87) |
| 2014 | 1.46(1.39–1.53) | 0.37(0.33–0.40) | 0.88(0.84–0.92) |
| 2015 | 1.52(1.45–1.60) | 0.40(0.36–0.43) | 0.94(0.90–0.98) |
| 2016 | 1.60(1.52–1.67) | 0.37(0.34–0.41) | 0.95(0.91–0.99) |
| 2017 | 1.61(1.54–1.69) | 0.36(0.33–0.39) | 0.96(0.93–1.00) |
| 2018 | 1.67(1.59–1.74) | 0.36(0.33–0.39) | 0.98(0.94–1.02) |
| 2019 | 1.65(1.58–1.73) | 0.40(0.37–0.43) | 1.00(0.96–1.04) |
| 2020 | 1.75(1.67–1.82) | 0.45(0.41–0.48) | 1.03(0.99–1.07) |
| 2021 | 1.89(1.81–1.96) | 0.39(0.36–0.42) | 1.08(1.04–1.12) |
| 2022 | 2.04(1.96–2.12) | 0.49(0.45–0.52) | 1.20(1.16–1.25) |
| 2023 | 2.00(1.93–2.08) | 0.44(0.40–0.47) | 1.17(1.13–1.21) |
| Total | 1.44(1.37–1.52) | 0.37(0.34–0.41) | 0.87(0.83–0.91) |

**Table 3. Annual Percent Change (APC) in Age-Adjusted Oropharyngeal Cancer Related Mortality Rates per 100,000 Among U.S. Adults by Sex, 1999–2023.**

| **Category** | **Year Interval** | **APC (95% CI)** | ***P*-value** |
| --- | --- | --- | --- |
| Overall | 1999–2009 | 0.42(-0.47–1.3) | 0.337758 |
|  | 2009–2023 | 3.02(2.59–3.46) * | < 0.000001 |
| Men | 1999–2009 | 0.37(-0.63–1.39) | 0.447067 |
|  | 2009–2023 | 3.45(2.98–3.92) * | < 0.000001 |
| Women | 1999–2017 | 0.21(-0.45–0.88) | 0.510435 |
|  | 2017–2023 | 3.92(0.82–7.11) * | 0.015495 |

* Indicates that the annual percent change (APC) is significantly different from zero at α = 0.05.

**Table 4. Average Annual percent change (AAPC) of Oropharyngeal Cancer Related Age-Adjusted Mortality Rates per 100,000 Among U.S. Adults by Sex, 1999–2023.**

| **Category** | **AAPC (95% CI)** |
| --- | --- |
| Both | 1.93(1.51–2.36) * |
| Female | 1.13(0.27–1.99) * |
| Male | 2.16(1.68–2.63) * |

* Indicates that the average annual percent change (AAPC) is significantly different from zero at α = 0.05.

**Table 5. Number of Oropharyngeal Cancer Deaths by Race and Year, 1999-2023.**

| **Year**  **NH White**  **NH Black**  **NH Other**  **Hispanic**  **Population** | **NH White** | **NH Black** | **NH Other** | **Hispanic** | **Population** |
| --- | --- | --- | --- | --- | --- |
| 1999  1999 | 1027 | 249 | 19 | 41 | 180408769 |
| 2000 | 957 | 223 | 20 | 44 | 181984640 |
| 2001 | 1098 | 258 | 23 | 56 | 184305128 |
| 2002 | 1118 | 233 | 27 | 52 | 186208028 |
| 2003 | 1107 | 236 | 29 | 57 | 188090429 |
| 2004 | 1156 | 231 | 28 | 59 | 190205384 |
| 2005 | 1175 | 241 | 29 | 60 | 192551384 |
| 2006 | 1162 | 245 | 24 | 62 | 195019359 |
| 2007 | 1256 | 266 | 27 | 67 | 197403777 |
| 2008 | 1326 | 251 | 24 | 55 | 199795090 |
| 2009 | 1374 | 239 | 21 | 60 | 202107016 |
| 2010 | 1488 | 258 | 24 | 75 | 203891983 |
| 2011 | 1518 | 289 | 32 | 91 | 206592936 |
| 2012 | 1571 | 280 | 39 | 94 | 208826037 |
| 2013 | 1548 | 251 | 41 | 103 | 211085314 |
| 2014 | 1737 | 297 | 37 | 104 | 213809280 |
| 2015 | 1887 | 266 | 54 | 121 | 216553817 |
| 2016 | 1970 | 296 | 61 | 109 | 218641417 |
| 2017 | 2021 | 297 | 60 | 149 | 221447331 |
| 2018 | 2104 | 310 | 51 | 144 | 223311190 |
| 2019 | 2179 | 321 | 71 | 151 | 224981167 |
| 2020 | 2361 | 336 | 63 | 157 | 226635013 |
| 2021 | 2472 | 318 | 82 | 172 | 228238412 |
| 2022 | 2794 | 351 | 101 | 179 | 229508599 |
| 2023 | 2790 | 309 | 101 | 166 | 231529762 |
| Total | 41196 | 6851 | 1088 | 2428 | 5163131262 |

**Table 6. Age-Adjusted Mortality Rates by Race and Year, 1999–2023.**

| **Year** | **NH White** | **NH Black** | **NH Other** | **Hispanic** |
| --- | --- | --- | --- | --- |
| 1999 | 0.72(0.67–0.76) | 1.49(1.30–1.67) | 0.36(0.19–0.52) | 0.40(0.28–0.56) |
| 2000 | 0.67(0.62–0.71) | 1.31(1.14–1.49) | 0.35(0.20–0.57) | 0.42(0.30–0.58) |
| 2001 | 0.74(0.70–0.79) | 1.45(1.27–1.62) | 0.32(0.20–0.50) | 0.47(0.35–0.62) |
| 2002 | 0.75(0.71–0.80) | 1.27(1.10–1.43) | 0.43(0.28–0.63) | 0.44(0.32–0.58) |
| 2003 | 0.73(0.68–0.77) | 1.32(1.15–1.49) | 0.42(0.27–0.62) | 0.45(0.34–0.59) |
| 2004 | 0.75(0.71–0.80) | 1.20(1.04–1.35) | 0.40(0.26–0.59) | 0.45(0.34–0.59) |
| 2005 | 0.73(0.69–0.78) | 1.20(1.05–1.36) | 0.37(0.24–0.53) | 0.41(0.31–0.54) |
| 2006 | 0.71(0.67–0.75) | 1.20(1.04–1.35) | 0.29(0.18–0.44) | 0.43(0.33–0.56) |
| 2007 | 0.78(0.73–0.82) | 1.25(1.09–1.40) | 0.32(0.21–0.46) | 0.47(0.36–0.60) |
| 2008 | 0.80(0.75–0.84) | 1.17(1.02–1.31) | 0.26(0.16–0.40) | 0.32(0.24–0.42) |
| 2009 | 0.82(0.78–0.86) | 1.09(0.95–1.24) | 0.25(0.15–0.38) | 0.33(0.25–0.43) |
| 2010 | 0.86(0.81–0.90) | 1.09(0.96–1.23) | 0.26(0.16–0.39) | 0.40(0.31–0.50) |
| 2011 | 0.86(0.82–0.90) | 1.22(1.08–1.37) | 0.30(0.20–0.43) | 0.47(0.37–0.58) |
| 2012 | 0.88(0.84–0.93) | 1.16(1.02–1.30) | 0.36(0.25–0.50) | 0.46(0.37–0.57) |
| 2013 | 0.87(0.82–0.91) | 1.06(0.92–1.19) | 0.36(0.25–0.49) | 0.48(0.38–0.57) |
| 2014 | 0.95(0.91–1.00) | 1.12(0.99–1.25) | 0.28(0.19–0.39) | 0.46(0.37–0.56) |
| 2015 | 1.02(0.97–1.07) | 0.99(0.87–1.12) | 0.39(0.29–0.51) | 0.51(0.41–0.60) |
| 2016 | 1.03(0.99–1.08) | 1.09(0.97–1.22) | 0.44(0.33–0.57) | 0.44(0.35–0.53) |
| 2017 | 1.03(0.99–1.08) | 1.09(0.96–1.22) | 0.41(0.31–0.53) | 0.57(0.47–0.67) |
| 2018 | 1.09(1.04–1.13) | 1.08(0.95–1.20) | 0.33(0.24–0.44) | 0.53(0.44–0.62) |
| 2019 | 1.10(1.05–1.14) | 1.10(0.98–1.23) | 0.46(0.35–0.58) | 0.54(0.45–0.63) |
| 2020 | 1.19(1.14–1.24) | 1.14(1.01–1.26) | 0.37(0.28–0.48) | 0.55(0.46–0.64) |
| 2021 | 1.25(1.20–1.30) | 1.08(0.96–1.21) | 0.43(0.34–0.54) | 0.58(0.48–0.67) |
| 2022 | 1.39(1.34–1.45) | 1.20(1.07–1.33) | 0.50(0.40–0.60) | 0.58(0.49–0.67) |
| 2023 | 1.36(1.31–1.41) | 1.02(0.90–1.14) | 0.48(0.39–0.58) | 0.51(0.43–0.59) |
| Total | 0.92(0.88–0.97) | 1.18(1.03–1.32) | 0.36(0.24–0.49) | 0.47(0.36–0.57) |

**Table 7. Annual percent change (APC) of Oropharyngeal Cancer Related Age-Adjusted Mortality Rates per 100,000 Among U.S. Adults by Race, 1999–2023.**

| **Category** | **Year Interval** | **APC (95% CI)** | ***P*-value** |
| --- | --- | --- | --- |
| NH White | 1999–2012 | 1.74(1.06–2.43) * | 0.000032 |
|  | 2012–2023 | 4.34(3.64–5.04) * | < 0.000001 |
| NH Black | 1999–2009 | -2.40(-3.67–-1.11) * | 0.000985 |
|  | 2009–2023 | -0.19(-0.92–0.54) | 0.588447 |
| NH Other | 1999–2009 | -3.53(-7.28–0.38) | 0.073581 |
|  | 2009–2023 | 3.85(2.25–5.47) * | 0.000058 |
| Hispanic | 1999–2023 | 1.56(0.93–2.19) * | 0.000032 |

* Indicates that the annual percent change (APC) is significantly different from zero at α = 0.05.

**Table 8. Average Annual percent change (AAPC) of Oropharyngeal Cancer Related Age-Adjusted Mortality Rates per 100,000 Among U.S. Adults by Race, 1999–2023.**

| **Category** | **AAPC (95% CI)** |
| --- | --- |
| Hispanic | 1.56(0.93–2.19) * |
| NH Black | -1.12(-1.76–-0.47) * |
| NH White | 2.92(2.46–3.39) * |
| NH Other | 0.71(-1.06–2.51) |

* Indicates that the average annual percent change (AAPC) is significantly different from zero at α = 0.05.

**Table 9. Number of Oropharyngeal Cancer Deaths by Census Region and Year, 1999–2023.**

| **Year**  **NH White**  **NH Black**  **NH Other**  **Hispanic**  **Population** | **Northeast** | **Midwest** | **South** | **West** | **Population** |
| --- | --- | --- | --- | --- | --- |
| 1999 | 268 | 310 | 501 | 262 | 180408769 |
| 2000 | 237 | 294 | 500 | 223 | 181984640 |
| 2001 | 295 | 273 | 596 | 277 | 184305128 |
| 2002 | 245 | 304 | 567 | 316 | 186208028 |
| 2003 | 256 | 316 | 570 | 291 | 188090429 |
| 2004 | 300 | 326 | 585 | 266 | 190205384 |
| 2005 | 279 | 330 | 609 | 290 | 192551384 |
| 2006 | 263 | 351 | 599 | 285 | 195019359 |
| 2007 | 267 | 382 | 663 | 308 | 197403777 |
| 2008 | 276 | 399 | 674 | 309 | 199795090 |
| 2009 | 294 | 373 | 694 | 339 | 202107016 |
| 2010 | 312 | 411 | 755 | 372 | 203891983 |
| 2011 | 310 | 449 | 785 | 390 | 206592936 |
| 2012 | 327 | 471 | 779 | 415 | 208826037 |
| 2013 | 338 | 422 | 787 | 404 | 211085314 |
| 2014 | 340 | 503 | 879 | 455 | 213809280 |
| 2015 | 368 | 540 | 910 | 521 | 216553817 |
| 2016 | 389 | 544 | 1025 | 491 | 218641417 |
| 2017 | 372 | 572 | 1029 | 559 | 221447331 |
| 2018 | 399 | 620 | 1067 | 534 | 223311190 |
| 2019 | 415 | 661 | 1121 | 529 | 224981167 |
| 2020 | 437 | 711 | 1176 | 602 | 226635013 |
| 2021 | 480 | 705 | 1252 | 613 | 228238412 |
| 2022 | 561 | 814 | 1401 | 660 | 229508599 |
| 2023 | 508 | 793 | 1359 | 715 | 231529762 |
| Total | 8536 | 11874 | 20883 | 10426 | 5163131262 |

**Table 10. Age-Adjusted Mortality Rates by Census Region and Year, 1999–2023.**

| **Year** | **Northeast** | **Midwest** | **South** | **West** |
| --- | --- | --- | --- | --- |
| 1999 | 0.74(0.65–0.83) | 0.75(0.66–0.83) | 0.80(0.73–0.87) | 0.73(0.65–0.82) |
| 2000 | 0.66(0.58–0.75) | 0.70(0.62–0.78) | 0.77(0.70–0.84) | 0.60(0.52–0.68) |
| 2001 | 0.78(0.69–0.87) | 0.65(0.58–0.73) | 0.92(0.85–0.99) | 0.71(0.63–0.80) |
| 2002 | 0.64(0.56–0.72) | 0.70(0.62–0.78) | 0.85(0.78–0.92) | 0.83(0.73–0.92) |
| 2003 | 0.66(0.58–0.74) | 0.72(0.64–0.81) | 0.83(0.76–0.89) | 0.73(0.65–0.82) |
| 2004 | 0.78(0.69–0.87) | 0.73(0.65–0.81) | 0.82(0.75–0.89) | 0.65(0.57–0.73) |
| 2005 | 0.69(0.61–0.78) | 0.74(0.66–0.82) | 0.83(0.76–0.89) | 0.69(0.61–0.77) |
| 2006 | 0.65(0.57–0.73) | 0.76(0.68–0.84) | 0.79(0.73–0.86) | 0.66(0.58–0.73) |
| 2007 | 0.66(0.58–0.74) | 0.81(0.73–0.89) | 0.89(0.82–0.96) | 0.68(0.61–0.76) |
| 2008 | 0.65(0.57–0.73) | 0.84(0.76–0.92) | 0.85(0.79–0.92) | 0.65(0.58–0.73) |
| 2009 | 0.70(0.62–0.78) | 0.74(0.67–0.82) | 0.84(0.78–0.91) | 0.73(0.65–0.80) |
| 2010 | 0.73(0.65–0.81) | 0.83(0.75–0.91) | 0.92(0.85–0.98) | 0.78(0.70–0.87) |
| 2011 | 0.72(0.64–0.81) | 0.87(0.78–0.95) | 0.90(0.84–0.97) | 0.78(0.70–0.86) |
| 2012 | 0.74(0.66–0.82) | 0.92(0.83–1.00) | 0.91(0.84–0.97) | 0.82(0.74–0.90) |
| 2013 | 0.75(0.67–0.83) | 0.79(0.71–0.86) | 0.88(0.81–0.94) | 0.78(0.70–0.86) |
| 2014 | 0.73(0.65–0.81) | 0.94(0.85–1.02) | 0.95(0.89–1.02) | 0.84(0.77–0.92) |
| 2015 | 0.80(0.71–0.88) | 0.97(0.89–1.05) | 0.98(0.92–1.05) | 0.92(0.84–1.00) |
| 2016 | 0.81(0.73–0.89) | 0.97(0.88–1.05) | 1.06(1.00–1.13) | 0.85(0.77–0.93) |
| 2017 | 0.76(0.68–0.84) | 1.01(0.93–1.10) | 1.04(0.98–1.11) | 0.93(0.85–1.01) |
| 2018 | 0.79(0.71–0.87) | 1.09(1.01–1.18) | 1.04(0.98–1.11) | 0.89(0.81–0.97) |
| 2019 | 0.85(0.76–0.93) | 1.14(1.05–1.23) | 1.09(1.02–1.15) | 0.87(0.79–0.95) |
| 2020 | 0.88(0.79–0.96) | 1.19(1.10–1.28) | 1.12(1.05–1.19) | 0.96(0.88–1.04) |
| 2021 | 0.95(0.86–1.04) | 1.18(1.09–1.26) | 1.20(1.14–1.27) | 0.99(0.91–1.07) |
| 2022 | 1.08(0.99–1.17) | 1.36(1.26–1.45) | 1.29(1.22–1.36) | 1.02(0.95–1.10) |
| 2023 | 0.96(0.87–1.04) | 1.28(1.19–1.37) | 1.25(1.18–1.31) | 1.08(1.00–1.16) |
| Total | 0.77(0.68–0.85) | 0.91(0.82–0.99) | 0.95(0.89–1.02) | 0.81(0.73–0.89) |

**Table 11. Annual percent change (APC) of Oropharyngeal Cancer Related Age-Adjusted Mortality Rates per 100,000 Among U.S. Adults by Census Region, 1999–2023.**

| **Category** | **Year Interval** | **APC (95% CI)** | **P-value** |
| --- | --- | --- | --- |
| Northeast region | 1999–2008 | -1.18(-3.16–0.84) | 0.235217 |
|  | 2008–2023 | 2.69(1.89–3.50) * | 0.000001 |
| South region | 1999–2013 | 0.85(0.21–1.50) * | 0.011722 |
|  | 2013–2023 | 3.48(2.64–4.32) * | < 0.000001 |
| Midwest region | 1999–2013 | 1.77(1.08–2.47) * | 0.000029 |
|  | 2013–2023 | 4.35(3.46–5.26) * | < 0.000001 |
| West region | 1999–2006 | -0.98(-3.84–1.97) | 0.491929 |
|  | 2006–2023 | 2.60(1.99–3.21) * | < 0.000001 |

* Indicates that the annual percent change (APC) is significantly different from zero at α = 0.05.

**Table 12. Average Annual percent change (AAPC) of Oropharyngeal Cancer Related Age-Adjusted Mortality Rates per 100,000 Among U.S. Adults by Census Region, 1999–2023.**

| **Category** | **AAPC (95% CI)** |
| --- | --- |
| Northeast | 1.22(0.37–2.09) * |
| Midwest | 2.84(2.32–3.36) * |
| South | 1.94(1.46–2.42) * |
| West | 1.54(0.64–2.46) * |

* Indicates that the average annual percent change (AAPC) is significantly different from zero at α = 0.05.

**Table 13. Number of Oropharyngeal Cancer Deaths by Urbanization and Year, 1999–2023.**

| **Year** | **Metropolitan** | **Nonmetropolitan** | **Population** |
| --- | --- | --- | --- |
| 1999 | 1123 | 218 | 180408769 |
| 2000 | 1066 | 188 | 181984640 |
| 2001 | 1189 | 252 | 184305128 |
| 2002 | 1213 | 219 | 186208028 |
| 2003 | 1199 | 234 | 188090429 |
| 2004 | 1232 | 245 | 190205384 |
| 2005 | 1254 | 254 | 192551384 |
| 2006 | 1192 | 306 | 195019359 |
| 2007 | 1311 | 309 | 197403777 |
| 2008 | 1349 | 309 | 199795090 |
| 2009 | 1381 | 319 | 202107016 |
| 2010 | 1535 | 315 | 203891983 |
| 2011 | 1580 | 354 | 206592936 |
| 2012 | 1603 | 389 | 208826037 |
| 2013 | 1584 | 367 | 211085314 |
| 2014 | 1778 | 399 | 213809280 |
| 2015 | 1938 | 401 | 216553817 |
| 2016 | 1993 | 456 | 218641417 |
| 2017 | 2091 | 441 | 221447331 |
| 2018 | 2138 | 482 | 223311190 |
| 2019 | 2225 | 501 | 224981167 |
| 2020 | 2405 | 521 | 226635013 |
| Total | 34379 | 7479 | 4473854489 |

**Table 14. Age-Adjusted Mortality Rates by Urbanization, 1999–2023.**

| **Year** | **Metropolitan** | **Nonmetropolitan** |
| --- | --- | --- |
| 1999 | 0.79(0.74–0.83) | 0.68(0.59–0.77) |
| 2000 | 0.72(0.68–0.77) | 0.59(0.51–0.68) |
| 2001 | 0.77(0.72–0.81) | 0.75(0.66–0.85) |
| 2002 | 0.77(0.73–0.82) | 0.65(0.57–0.74) |
| 2003 | 0.75(0.71–0.80) | 0.70(0.61–0.78) |
| 2004 | 0.77(0.72–0.81) | 0.70(0.61–0.79) |
| 2005 | 0.77(0.72–0.81) | 0.73(0.64–0.82) |
| 2006 | 0.70(0.66–0.74) | 0.84(0.75–0.94) |
| 2007 | 0.74(0.70–0.78) | 0.83(0.73–0.92) |
| 2008 | 0.77(0.73–0.81) | 0.81(0.72–0.90) |
| 2009 | 0.77(0.73–0.81) | 0.82(0.73–0.91) |
| 2010 | 0.83(0.78–0.87) | 0.83(0.74–0.92) |
| 2011 | 0.84(0.79–0.88) | 0.88(0.79–0.98) |
| 2012 | 0.82(0.78–0.86) | 0.99(0.89–1.09) |
| 2013 | 0.80(0.76–0.85) | 0.92(0.82–1.01) |
| 2014 | 0.87(0.83–0.91) | 0.98(0.88–1.08) |
| 2015 | 0.93(0.89–0.98) | 0.98(0.88–1.08) |
| 2016 | 0.93(0.89–0.97) | 1.08(0.98–1.19) |
| 2017 | 0.94(0.90–0.98) | 1.05(0.95–1.14) |
| 2018 | 0.95(0.91–1.00) | 1.13(1.03–1.23) |
| 2019 | 0.96(0.92–1.01) | 1.15(1.05–1.25) |
| 2020 | 1.03(0.99–1.08) | 1.19(1.09–1.30) |
| Total | 0.83(0.79–0.87) | 0.88(0.78–0.97) |

**Table 15. Annual percent change (APC) of Oropharyngeal Cancer Related Age-Adjusted Mortality Rates per 100,000 Among U.S. Adults by Urbanization, 1999–2023.**

| **Category** | **Year Interval** | **APC (95% CI)** | **P-value** |
| --- | --- | --- | --- |
| Nonmetropolitan areas Metropolitan area | 1999–2020 | 2.98(2.62–3.34) * | < 0.000001 |
| Metropolitan | 1999–2006 | -0.84(-2.07–3.40) | 0.16995 |
|  | 2009–2020 | 2.33(1.96–2.70) * | < 0.000001 |

* Indicates that the annual percent change (APC) is significantly different from zero at α = 0.05.

**Table 16. Average Annual percent change (AAPC) of Oropharyngeal Cancer Related Age-Adjusted Mortality Rates per 100,000 Among U.S. Adults by Urbanization, 1999–2023.**

| **Category** | **AAPC (95% CI)** |
| --- | --- |
| Metropolitan | 1.26(0.81–1.71) * |
| Nonmetropolitan | 2.98(2.62–3.34) * |

* Indicates that the average annual percent change (AAPC) is significantly different from zero at α = 0.05.

**Table 17. Total Number of Oropharyngeal Cancer Deaths by State, 1999–2023.**

| **State** | **Death_1999** | **Death_2023** | **Death_all** |
| --- | --- | --- | --- |
| Alabama | 26 | 45 | 827 |
| Arizona | 16 | 55 | 848 |
| California | 151 | 314 | 5134 |
| Florida | 112 | 275 | 4366 |
| Georgia | 42 | 105 | 1490 |
| Illinois | 61 | 133 | 2062 |
| Indiana | 25 | 68 | 1117 |
| Iowa | 17 | 33 | 541 |
| Kentucky | 20 | 55 | 859 |
| Louisiana | 19 | 50 | 832 |
| Maryland | 28 | 68 | 990 |
| Massachusetts | 34 | 73 | 1069 |
| Michigan | 49 | 117 | 1845 |
| Minnesota | 25 | 69 | 864 |
| Missouri | 21 | 76 | 1126 |
| New Jersey | 47 | 64 | 1087 |
| New York | 88 | 146 | 2826 |
| North Carolina | 33 | 130 | 1851 |
| Ohio | 65 | 161 | 2408 |
| Oklahoma | 13 | 35 | 591 |
| Oregon | 17 | 59 | 830 |
| Pennsylvania | 62 | 136 | 2234 |
| South Carolina | 32 | 79 | 1023 |
| Tennessee | 33 | 78 | 1322 |
| Texas | 78 | 238 | 3741 |
| Virginia | 31 | 73 | 1306 |
| Washington | 31 | 89 | 1334 |
| Wisconsin | 26 | 75 | 1087 |

**Table 18. Age-Adjusted Mortality Rates by State and Year, 1999–2023.**

| **State** | **AAMR_1999** | **AAMR_2023** | **AAMR** |
| --- | --- | --- | --- |
| Alabama | 0.87(0.57–1.27) | 0.98(0.71–1.32) | 0.90(0.59–1.21) |
| Arizona | 0.55(0.29–0.82) | 0.83(0.62–1.09) | 0.67(0.44–0.90) |
| California | 0.83(0.70–0.97) | 1.01(0.89–1.12) | 0.80(0.69–0.92) |
| Florida | 0.90(0.73–1.07) | 1.26(1.11–1.42) | 1.02(0.86–1.17) |
| Georgia | 0.91(0.65–1.24) | 1.21(0.97–1.44) | 0.90(0.66–1.13) |
| Illinois | 0.80(0.61–1.03) | 1.24(1.02–1.45) | 0.88(0.68–1.07) |
| Indiana | 0.66(0.43–0.97) | 1.15(0.89–1.46) | 0.89(0.63–1.16) |
| Iowa | 0.87(0.46–1.27) | 1.17(0.80–1.66) | 0.92(0.53–1.30) |
| Kentucky | 0.75(0.46–1.16) | 1.41(1.05–1.85) | 1.02(0.68–1.36) |
| Louisiana | 0.77(0.43–1.11) | 1.26(0.93–1.68) | 0.99(0.65–1.33) |
| Maryland | 0.86(0.57–1.24) | 1.28(0.99–1.62) | 0.92(0.62–1.21) |
| Massachusetts | 0.80(0.55–1.12) | 1.13(0.88–1.43) | 0.81(0.57–1.06) |
| Michigan | 0.79(0.58–1.04) | 1.23(1.00–1.46) | 0.93(0.71–1.15) |
| Minnesota | 0.83(0.54–1.22) | 1.42(1.10–1.80) | 0.84(0.56–1.13) |
| Missouri | 0.55(0.34–0.84) | 1.36(1.07–1.72) | 0.95(0.67–1.23) |
| New Jersey | 0.85(0.63–1.13) | 0.75(0.57–0.95) | 0.64(0.45–0.84) |
| New York | 0.71(0.57–0.88) | 0.82(0.68–0.95) | 0.76(0.62–0.90) |
| North Carolina | 0.69(0.47–0.96) | 1.38(1.14–1.62) | 1.01(0.77–1.25) |
| Ohio | 0.87(0.67–1.11) | 1.50(1.26–1.74) | 1.03(0.82–1.24) |
| Oklahoma | 0.64(0.30–0.98) | 1.05(0.73–1.46) | 0.86(0.52–1.20) |
| Oregon | 0.83(0.44–1.22) | 1.48(1.12–1.91) | 1.06(0.70–1.42) |
| Pennsylvania | 0.69(0.53–0.89) | 1.05(0.87–1.23) | 0.83(0.66–1.01) |
| South Carolina | 1.28(0.87–1.80) | 1.61(1.26–2.01) | 1.12(0.77–1.47) |
| Tennessee | 0.89(0.62–1.25) | 1.29(1.01–1.61) | 1.07(0.78–1.37) |
| Texas | 0.71(0.56–0.89) | 1.10(0.96–1.25) | 0.90(0.75–1.05) |
| Virginia | 0.71(0.48–1.01) | 0.99(0.77–1.25) | 0.86(0.62–1.10) |
| Washington | 0.89(0.60–1.26) | 1.41(1.13–1.74) | 1.06(0.77–1.35) |
| Wisconsin | 0.74(0.48–1.09) | 1.40(1.09–1.76) | 0.96(0.67–1.25) |

**Table 19. Average Annual percent change (AAPC) of Oropharyngeal Cancer Related Age-Adjusted Mortality Rates per 100,000 in State in the United States, 1999–2023.**

| **State** | **AAPC (95% CI)** |
| --- | --- |
| Alabama | 1.30(0.43–2.19) |
| Arizona | 2.63(1.89–3.37) * |
| California | 1.06(0.24–1.90) * |
| Florida | 1.11(0.48–1.75) * |
| Georgia | 1.78(0.87–2.69) * |
| Illinois | 2.15(1.50–2.80) * |
| Indiana | 3.92(2.77–5.09) * |
| Iowa | 1.35(0.31–2.40) * |
| Kentucky | 3.30(2.19–4.42) * |
| Louisiana | 2.02(1.05–3.00) * |
| Maryland | 1.39(-0.09–2.88) |
| Massachusetts | 1.50(0.54–2.47) * |
| Michigan | 2.13(-2.22–6.67) |
| Minnesota | 1.89(0.02–3.79) * |
| Missouri | 3.58(2.72–4.44) * |
| New Jersey | -0.10(-1.35–1.16) |
| New York | 0.70(0.14–1.27) * |
| North Carolina | 2.87(2.12–3.63) * |
| Ohio | 3.14(2.50–3.78) * |
| Oklahoma | 2.16(0.72–3.63) * |
| Oregon | 3.03(1.74–4.34) * |
| Pennsylvania | 2.71(1.99–3.44) * |
| South Carolina | 0.88(-0.92–2.72) |
| Tennessee | 1.77(0.90–2.65) * |
| Texas | 2.11(1.57–2.66) * |
| Virginia | 2.50(1.70–3.31) * |
| Washington | 1.75(0.84–2.66) * |
| Wisconsin | 2.83(2.00–3.67) * |

* Indicates that the average annual percent change (AAPC) is significantly different from zero at α = 0.05.

**Table 20. Summary of Average AAMR (2019–2023), Poverty rates, Dentist density, and HPV Vaccination rates by State.**

| **state** | **AAMR(2019-2023)** | **Poverty rate** | **Dentist density** | **HPV rate** |
| --- | --- | --- | --- | --- |
| Alabama | 1.01 | 17.53 | 43.22 | 44 |
| Arizona | 0.86 | 18.52 | 53.17 | 53.8 |
| California | 0.95 | 15.68 | 76.55 | 46.6 |
| Florida | 1.18 | 16.9 | 50.75 | 32.2 |
| Georgia | 1.13 | 17.37 | 46.03 | 40.4 |
| Illinois | 1.12 | 14.52 | 65.82 | 33.1 |
| Indiana | 1.25 | 15 | 46.9 | 29.3 |
| Iowa | 0.96 | 12.52 | 52.43 | 42.9 |
| Kentucky | 1.36 | 18.92 | 54.1 | 38.8 |
| Louisiana | 1.27 | 18.48 | 47.3 | 52.8 |
| Maryland | 1.13 | 10.3 | 71.55 | 45.8 |
| Massachusetts | 1.01 | 12.33 | 81.2 | 54.7 |
| Michigan | 1.27 | 16.05 | 60.47 | 39.4 |
| Minnesota | 1.19 | 13.25 | 59.95 | 42.6 |
| Missouri | 1.3 | 15.53 | 48.85 | 33.3 |
| New Jersey | 0.73 | 12.4 | 81.37 | 46.3 |
| New York | 0.82 | 15.85 | 75.63 | 42.9 |
| North Carolina | 1.31 | 16.52 | 48.77 | 34.1 |
| Ohio | 1.46 | 16 | 52 | 33.8 |
| Oklahoma | 1.1 | 16.65 | 48.03 | 35.6 |
| Oregon | 1.38 | 16.07 | 66.55 | 46 |
| Pennsylvania | 1.13 | 13.68 | 60.63 | 46.9 |
| South Carolina | 1.26 | 17.6 | 46.52 | 33.3 |
| Tennessee | 1.4 | 17.65 | 48.68 | 35.3 |
| Texas | 1.12 | 16.85 | 49.05 | 38.2 |
| Virginia | 1.06 | 11.72 | 61.18 | 46.8 |
| Washington | 1.22 | 14.18 | 69.7 | 52.9 |
| Wisconsin | 1.32 | 12.88 | 56.54 | 42.9 |

**Table 21. Total Number of Oropharyngeal Cancer Deaths by Age and Year, 1999–2023.**

| **Age group** | **Total death number** |
| --- | --- |
| 35–44 years | 1048 |
| 45–54 years | 7071 |
| 55–64 years | 15599 |
| 65–74 years | 14940 |
| 75–84 years | 9364 |
| 85+ years | 3644 |

**Table 22. Crude Mortality Rates by Age Group, 1999–2023.**

| **Age group** | **CMR** |
| --- | --- |
| 35–44 years | 0.10(0.07–0.13) |
| 45–54 years | 0.67(0.59–0.75) |
| 55–64 years | 1.70(1.57–1.84) |
| 65–74 years | 2.32(2.13–2.52) |
| 75–84 years | 2.60(2.34–2.87) |
| 85+ years | 2.58(2.16–3.00) |

**Table 23. Annual percent change (APC) of Oropharyngeal Cancer Related Age-Adjusted Mortality Rates per 100,000 Among U.S. Adults by Age Group, 1999–2023.**

| **Category** | **Year Interval** | **APC (95% CI)** | **P-value** |
| --- | --- | --- | --- |
| 35-44 years | 1999–2023 | -1.69(-2.47–-0.90) * | 0.000195 |
| 45-54 years | 1999–2023 | -0.03(-0.40–0.35) | 0.874862 |
| 55-64 years | 1999–2006 | -0.22(-2.35–1.96) | 0.835474 |
|  | 2006–2023 | 2.78(2.35–3.21) * | < 0.000001 |
| 65-74 years | 1999–2008 | -0.51(-1.97–0.97) | 0.47533 |
|  | 2008–2023 | 4.20(3.69–4.72) * | < 0.000001 |
| 75-84 years | 1999–2016 | 1.72(0.94–2.51) * | 0.000177 |
|  | 2016–2023 | 6.89(4.58–9.26) * | 0.000003 |
| 85+ years | 1999–2013 | 0.81(-0.44–2.08) | 0.194318 |
|  | 2013–2023 | 4.92(3.32–6.54) * | 0.000002 |

* Indicates that the annual percent change (APC) is significantly different from zero at α = 0.05.

**Table 24. Average Annual percent change (AAPC) of Oropharyngeal Cancer Related Age-Adjusted Mortality Rates per 100,000 Among U.S. Adults by Age Group, 1999–2023.**

| **Category** | **AAPC (95% CI)** |
| --- | --- |
| 35–44 years | -1.69(-2.47–-0.90) * |
| 45–54 years | -0.03(-0.40–0.35) |
| 55–64 years | 1.90(1.23–2.57) * |
| 65–74 years | 2.41(1.80–3.02) * |
| 75–84 years | 3.20(2.39–4.02) * |
| 85+ years | 2.50(1.57–3.44) * |

* Indicates that the average annual percent change (AAPC) is significantly different from zero at α = 0.05.
